# Advances in Unhealthy Nutrition and Circadian Dysregulation in Pathophysiology of NAFLD

**DOI:** 10.3389/fcdhc.2021.691828

**Published:** 2021-10-12

**Authors:** Xin Guo, Juan Zheng, Shixiu Zhang, Xiaofan Jiang, Ting Chen, Jiayu Yu, Shu'e Wang, Xiaomin Ma, Chaodong Wu

**Affiliations:** ^1^ Department of Nutrition and Food Hygiene, School of Public Health, Cheeloo College of Medicine, Shandong University, Jinan, China; ^2^ Department of Endocrinology, Union Hospital, Tongji Medical College, Huazhong University of Science and Technology, Wuhan, China; ^3^ Hubei Provincial Clinical Research Center for Diabetes and Metabolic Disorders, Wuhan, China; ^4^ Department of Nutrition, Texas A&M University, College Station, TX, United States

**Keywords:** circadian, nutrition, metabolic diseases, NAFLD, hepatic steatosis, inflammation

## Abstract

Unhealthy diets and lifestyle result in various metabolic conditions including metabolic syndrome and non-alcoholic fatty liver disease (NAFLD). Much evidence indicates that disruption of circadian rhythms contributes to the development and progression of excessive hepatic fat deposition and inflammation, as well as liver fibrosis, a key characteristic of non-steatohepatitis (NASH) or the advanced form of NAFLD. In this review, we emphasize the importance of nutrition as a critical factor in the regulation of circadian clock in the liver. We also focus on the roles of the rhythms of nutrient intake and the composition of diets in the regulation of circadian clocks in the context of controlling hepatic glucose and fat metabolism. We then summarize the effects of unhealthy nutrition and circadian dysregulation on the development of hepatic steatosis and inflammation. A better understanding of how the interplay among nutrition, circadian rhythms, and dysregulated metabolism result in hepatic steatosis and inflammation can help develop improved preventive and/or therapeutic strategies for managing NAFLD.

## Introduction

With the ongoing pandemic of obesity, 25% of the population worldwide, including children, adolescents and adults, are suffering from non-alcoholic fatty liver disease (NAFLD) (1). NAFLD is characterized by excess accumulation of triglycerides in the hepatocytes (hepatic steatosis) due to both increased inflow of free fatty acids and *de novo* lipogenesis. When the liver exhibits overt inflammatory damage and fibrosis, NAFLD progresses to its advanced stage, nonalcoholic steatohepatitis (NASH). The latter has increased risk to progress to liver cirrhosis and hepatocellular carcinoma. Metabolic abnormalities related to unhealthy nutrition, such as central obesity, insulin resistance, dyslipidemia and hypertension, are closely related to NAFLD (2). Although the etiology and progression of NAFLD remain to be elucidated, more and more studies have shown that the circadian clocks play a key role in the regulation of key aspects of the pathogenesis of NAFLD (3, 4).

Circadian rhythms are widely present in animals and plants. In mammals, circadian rhythms function to coordinate a diverse panel of physiological processes that are influenced by environmental rhythmic signals such as food and light. In addition, circadian dysfunction is associated with sleep disorders, elevated incidence of cancer, and metabolic abnormalities. Located in the suprachiasmatic nucleus (SCN) of the hypothalamus, the master clock plays an important role in governing biological rhythms, coordinating the peripheral clocks in peripheral tissues such as the liver, muscle, adipose tissue and gastrointestinal tract. The master clock receives light and generates timing signals to govern peripheral clocks, shaping whole body’s circadian rhythms. Also, the master clock drives circadian rhythms of behavior such as rest and activity cycles while peripheral clocks play more important roles in physiological regulation of each peripheral tissue (5, 6).

Recent studies have indicated that the misalignment of the central clock and the peripheral clock results in dysregulated metabolism of glucose and fat. Indeed, abnormal rhythmic cycles are related to metabolic diseases such as obesity, diabetes and NAFLD (7). Of note, the liver is considered a clock organ because many genes related to metabolism in the liver exhibit diurnal rhythmicity and are regulated by the circadian clock.

## Description of the Molecular Clock

A large number of physiological events follow circadian rhythmicity. At the cellular level, circadian rhythms are generated by an auto regulatory transcriptional and translational feedback loop (TTFL) consisting of the clock genes Period (PER) 1, PER2, Cryptochrome (CRY) 1, CRY2, brain and muscle aryl hydrocarbon receptor nuclear translocator-like protein 1 (BMAL1), circadian locomotor output cycles kaput (CLOCK) and their protein products (8–12). In the core loop of the mammalian clocks, the CLOCK: BMAL1 heterodimer binds to enhancer elements (E-boxes) to initiate the transcription of PER, CRY and other clock-controlled genes (CCGs). PER: CRY dimers accumulate in the nucleus over the course of the day to inhibit the activity of the CLOCK: BMAL1 heterodimer, thereby suppressing their own transcription. PER and CRY also can be broken down by β-transducing repeat-containing protein (β–TrCP) and F-box/LRR-repeat protein 3 (FBXL3) respectively to reset the cycle. In addition, the CLOCK: BMAL1 heterodimer also regulates the transcription of REV-ERBs, which are nuclear receptors and compete with retinoic acid-related orphan receptors (RORs) to inhibit the transcription of BMAL1 and delay the transcription of CRY1 (5, 13, 14).

Coordination at the cellular level is necessary for tissue-specific oscillations that regulate circadian physiology (15–17) and the alignment of these clocks between tissues is essential for the maintenance of metabolic homeostasis. The possibility of misalignment arises from the differential responsiveness of tissues to the environmental cues that synchronize the clock (zeitgebers). Although light is the dominant environmental rhythmic cues that resents the master clock of the SCN, many other tissues are sensitive to cues derived from nutritional rhythms (18).

The liver is involved in the decomposition of three major macronutrients. About one tenth of the liver transcriptome are expressed rhythmically (19). In the liver of wild-type (WT) animals, the rhythm of a CLOCK: BMAL1 is coordinated during the day, accompanied with histone acetylation and RNA polymerase II accumulation, which triggers the peak of transcription in the early phase of night (16, 20). While the translation of inhibitory CRY and PER proteins increases, the levels of CLOCK and BMAL1 decrease and CLOCK:BMAL1-driven transcription is inhibited (20). This forms a core feedback inhibitory loop of circadian clock. REV-ERBα, also functioning to adjust CLOCK: BMAL1, reaches its peak expression at ZT8-10 in the liver, and returns to the lowest levels at ZT20-22. This constitutes another negative feedback loop (14, 21). However, it should be noted that only a very small number (less than 10%) of rhythmic genes are common to the liver, kidney and heart whereas most of rhythmic genes are tissue-specific (22, 23). Upon reconstituting circadian expression of BMAL1 only in the liver of BMAL1-/- mice, the liver recovered about 10% of total hepatic rhythmic transcripts and 20% of oscillatory metabolites, including oscillation for glycogen and NAD^+^ salvage metabolism (16).

Energy metabolism and nutrient absorption are integrated with the peripheral clock of the liver (24). The amplitude of BMAL1, a critical driver of metabolic homeostasis under physiological conditions, is decreased in light and dark periods in response to high-fat feeding, resulting in the dysregulation of oscillation rhythms (25, 26). Global disruption of BMAL1 in mice exhibited increased body weight and fat content and decreased insulin secretion. When disrupting BMAL1 only in the liver, mice did not show differences in body weight, fat content and serum insulin levels compared to wild type mice. However, the rhythmic expression of clock-regulated metabolic genes such as glucose transporter 2 (GLUT2), glucokinase, liver pyruvate kinase, phosphoenolpyruvate carboxykinase 1 (PEPCK1), carnitine palmitoyltransferase 1 (CPT1), etc. are abolished. In addition, liver-specific disruption of BMAL1 increased glucose clearance and induced hypoglycemia (27).

Metabolism in the liver is closely synchronized with the clock, making the pace of demand and supply consistent. For example, upon food consumption after late-night fasting during sleep, circadian clock provides rhythmic and baseline regulation and repetitive activities, resulting in rapid consumption of glucose in the morning to meet energy demand (4). Furthermore, the liver acts as a transit point for toxins. For example, amino acids (exogenous substances) are converted to ammonia (transformation) and then urea (water soluble metabolites) in the liver, which is a well-known process of detoxification. The first step of transformation involves hepatic nuclear receptors which are controlled by rhythmic expression, such as REV-ERB and ROR subfamilies (28). Core clock and clock-controlled genes regulate hepatic glucose metabolism. For example, in the liver, glucagon stimulates gluconeogenesis by activating cAMP/cAMP-response element binding protein (CREB) signaling during fasting. PER inhibits glucagon-stimulated cAMP production to decrease gluconeogenesis via interacting with G protein–coupled receptors (29). Peroxisome proliferator-activated receptor (PPAR), which is a family of nuclear receptor proteins serving as the transcription factors to regulate gene expression related to lipid metabolism, is controlled by the circadian clock. Hepatic PPARα and PPARγ are activated during day time and are inhibited during night time, while hepatic PPARδ is induced during night time (28). REV-ERBα modulates the oscillation of sterol regulatory element-binding protein (SREBP) activity, which is involved in cholesterol and lipid metabolism. In addition, REV-ERBα also is involved in the circadian transcription of cholesterol-7a-hydroxylase (CYP7A1), which is the key enzyme in bile acid synthesis (30).

Predictably, any factor that alters circadian rhythms can cause hepatic metabolic dysregulation, in the course of time, leading to metabolic diseases. In the liver, circadian mRNA and protein expression of clock genes such as PER1, PER2, BMAL1 and CLOCK and circadian-related metabolic regulators, such as AMPK, lipogenic enzymes, and gluconeogenic proteins are changed in response to HFD feeding, leading to obesity and insulin resistance (29, 31). Based on this, it is conceivable that circadian rhythms control various metabolic processes in the liver. When the phases of metabolic genes are misaligned, diseases and disorders often occur.

## Nutrient Control of the Circadian Clock

Nutrient supply and circadian rhythms are intimately linked. Feeding a high fat diet (HFD) disrupts circadian rhythms and causes an unexpectedly large-scale genesis of *de novo* oscillating transcripts, resulting in reorganization of the coordinated oscillations between transcripts and metabolites. The mechanisms underlying this reprogramming involve both the impairment of CLOCK: BMAL1 chromatin recruitment and a pronounced cyclic activation of surrogate pathways through the transcriptional regulator peroxisome proliferator-activated receptor γ (PPARγ) (32). An *in vivo* study revealed that it takes only 3 days for an HFD to change the circadian clock in the liver (20). Moreover, HFD feeding for 4 weeks significantly altered the rhythms of fatty acid synthesis rate-limiting enzyme hepatic acetyl-CoA carboxylase (ACACA), REV-ERBα and histone regulator HDAC3 (33). Studies have shown that insulin fluctuations caused by nutrient supply can “reset” the biological clock of the liver (34). In insulin-deficient mice, this change was not observed (35) but injection of insulin reset the rhythmicity of genes. This process involved phosphoinositide 3-kinase (PIK3) and mitogen-activated protein kinase (MAPK) pathways (36).

Conversely, food restriction can almost reset some peripheral clocks entirely (37–39). During fasting, increased phosphorylation of adenosine monophosphate (AMP)-activated protein kinase (AMPK) destroyed the abundance of CRY and targeted CRYI for its subsequent degradation, thus preventing CRY from inhibiting CLOCK: BMAL 1 target genes (40). The duration period of fasting also affects the peripheral biological clocks. Compared with short-term fasting, long-term fast is shown to cause a stronger stimulatory effect on the peripheral biological clock system (41, 42). Temporal feeding restriction under light-dark or dark-dark conditions is shown to change the phase of circadian gene expression in peripheral cell types by up to 12 h, while leaving the phase of rhythmic gene expression in the SCN unaffected. As such, changes in nutrient supply can uncouple peripheral oscillators and the central pacemaker. The persistence of circadian clock gene oscillation in both normal chow and HFD validates the notion that circadian oscillation within the core clock genes is highly resistant to perturbation, whereas clock output genes are more sensitive to food as a zeitgeber (39).

Compared with direct circadian control, nutrient rhythmic supply in the form of feeding/fating rhythms is an indirect but vital contributor of rhythmic hepatic transcripts (43). In general, the liver oscillator is controlled by signals from the SCN, and these signals are amplified by normal feeding rhythms (39). When feeding rhythm changes, central and peripheral rhythmic signals clash, leading to metabolic changes. Studies have shown that in rodents disordered feeding rhythms led to weight gain (44, 45). In human subjects, changes in meal schedules cause the central circadian rhythm to be out of sync with environmental signals, resulting in the circadian dysregulation (46). The molecular basis for this metabolic disruption likely is attributable to the dissociation of metabolic gene rhythms caused by inconsistency between the central clock and nutrient rhythms. Feeding a high-fat food in limited time does not change the overall caloric intake as a whole, but improved diurnal. This timed feeding in mice was shown to improve diurnal rhythms in metabolic regulators and the circadian oscillator, thereby improving insulin sensitivity and reducing adiposity in adipose tissue and liver, compared to mice fed with an HFD ad libitum (45). While exploring how nutrients couple clock regulation, two studies have demonstrated that the cellular pathways of NAD^+^ metabolism (47) and the concentrations of adenosine monophosphate (48) appear to link the availability of nutrients and rhythmic regulation. This led to the creation of the concept of chrononutrition (24), which aims to use timed nutrient supply as effective strategy to regulate circadian clocks in the liver (49, 50).

In addition to food intake rhythm, the nutrient composition of the food could also disrupt the normal outputs of body clock signals. For example, saturated fatty acids have been shown to alter the expression of clock genes in cell lines (51–53). Certain studies also have shown that in cell cultures, palmitate, the most common type of saturated fatty acid found in obese animals, disrupted protein–protein interaction between CLOCK and BMAL1 in a dose- and time-dependent manner. This inhibitory effect of palmitate was reversed by Sirtuin 1 (SIRT1) activator (52). In addition, docosahexaenoic acid (DHA), a polyunsaturated fatty acid (PUFA), functioned to alleviate the effect of palmitate on decreasing BMAL1 in cells; although DHA per se altered the circadian rhythm of BMAL1, suggesting a protective role (54, 55). Notably, the effect of saturated fat on the body clock has never been tested *in vivo*. A recent study suggested non-obesogenic doses of palmitate appeared to change circadian rhythms. In addition, non-obesogenic doses of oleate, a monounsaturated fatty acid (MUFA), also functioned to cause circadian dysregulation. Overall, saturated fatty acids are more harmful (56), although unsaturated fatty acids also alter circadian rhythms.

Additional to fats, amino acids also regulate liver clocks. For instance, the peak of liver PER2 is altered in response to an intravenous injection of mixture of 18 amino acids (57). Another recent study showed that feeding mice a diet containing only protein and/or amino acids changed liver circadian rhythms but not increased insulin levels, which is likely because pure protein diets and cysteine stimulated glucagon secretion and/or increased the production of insulin-like growth factor (IGF-1) (58). Collectively, the existing evidence indicates that altering food composition and the timing of nutrient supply appears to be a feasible approach for managing metabolic diseases. Of note, the effectiveness of nutritional regulation of the circadian clocks may vary depending on age and sex (15).

In short, nutrients regulate hepatic circadian rhythm through directly or indirectly modulating the expression of hepatic circadian-related genes. The investigation of hepatic circadian regulation by nutrition is expected to provide new knowledge concerning the mechanisms of liver metabolism and metabolic dysregulation-related liver diseases.

## Circadian Dysregulation in the Pathogenesis of NAFLD

The pathogenesis of NAFLD and NASH were illustrated largely by a “two hit hypothesis”. The “first hit” is characterized by fat accumulation in liver, leading to hepatic steatosis and liver inflammatory damage. The “second hit” is referred to the concept that the damaged liver is susceptible to proinflammatory cytokines, adipokines and oxidative stress, resulting in steatohepatitis and liver fibrosis (59). Much evidence has shown that circadian rhythmic dysregulation is related to the occurrence and the progression of NAFLD/NASH (60, 61). Therefore, circadian rhythm-related hepatic lipid metabolism and inflammatory response has attracted much attention with hope to better manage NAFLD.

### Circadian Dysregulation Promotes Hepatic Steatosis

In terms of the pathogenesis of NAFLD, the primary stage of NAFLD onset is excessive accumulation of triglycerides (TG) (59, 62). Fats depose in the liver with the following routes: 1) free fatty acids from adipose tissue lipolysis are transported to the liver. In most cases, insulin resistance results in an increase in lipolysis in the adipose tissue, leading to increased free fatty acids mobilization to the bloodstream and increased influx of free fatty acids to hepatocytes (63); 2) dietary fats digested and absorbed into lymph vessels by forming a lipoprotein called chylomicron. Some free fatty acids from chylomicron goes to deposit in adipose tissue, while other fatty acids are still in chylomicron remnants which are delivered to liver (64); 3) de novo lipogenesis is increased, especially by increasing dietary simple sugar (65); 4) fatty acid oxidation is decreased (66); 5) fatty acids, which derived from diets, de novo lipogenesis, and lipolysis of fats in adipose tissue are bound to glycerol to produce TG (67).

Nutritional signal (glucose) and hormonal signal (insulin) up-regulate carbohydrate responsive element-binding protein (ChREBP) and Sterol Regulatory Element-binding Protein-1c (SREBP-1c) *via* liver X receptors (LXRs) to increase the expression of genes for lipogenic enzymes such as acetyl-CoA carboxylase (ACC), fatty acid synthase (FASN), and stearoyl-CoA desaturase (SCD-1), thereby promoting hepatic lipid accumulation in the liver in response to feeding. Of note, the results from the studies involving multi-stable isotope labeling methods prove that fat formation is the key point in the process of hepatic steatosis (68, 69). It is now widely accepted that elevated TG formation in liver causes simple steatosis, and when liver lipotoxicity is overt, it has promoted the progression of simple steatosis to NASH. While the fatty acids used to synthesize TG are derived from diets, de novo lipogenesis, and lipolysis of fats in adipose tissue (66).

Excessive accumulation of fats is the key characteristic of NAFLD. Circadian disorders are involved in metabolic disruption, especially liver metabolic pathways (**Table 1**), which contribute to the development of NAFLD (3, 64, 76). According to the findings from a lipidomic analysis (3), TG is a significant component of oscillating lipids in the liver. Under normal circumstances, TG accumulate and disappear quantitatively every day. X-box binding protein 1 (XBP1) regulated the hepatic 12-h cistrome and was found to adjust TG transport and the levels of VLDL-TG (77). Disruption of XBP1 promoted NAFLD development, likely through altering the temporal 12-h transcription of lecithin–cholesterol acyltransferase, lysophosphatidylcholine acyltransferase 3, and stearoyl-CoA desaturase 1, and through impairing phosphatidylcholine and lysophosphatidylcholines cycle, as well as fatty acid monounsaturation (73). Differentiated embryo-chondrocyte expressed gene 1 (DEC1), which is a regulator of the circadian clock, inhibited the expression of SREBP-1c to reduce hepatic lipogenesis and ameliorated fatty liver phenotype in NAFLD mouse models (70). In rat primary hepatocytes, circadian transcriptional regulators such as DEC1 and Kruppel-like factor-10 (KLF-10) formed a feedback loop and were involved in the regulation of hepatic lipogenesis *via* ChREBP (71, 72). Moreover, both cytochrome P450 and 3-hydroxy-3-methylglutaryl coenzyme A (HMG CoA) enzymes are involved in the synthesis and decomposition of fats as rate limiting enzymes with circadian rhythms (78). For example, cholesterol 7-alpha-monooxygenase (CYP7A1), which is a cytochrome P450 enzyme in cholesterol metabolism, was largely upregulated in patients with NAFLD (79). CYP7A1 was also reported to control circadian clock in the liver. Inhibition of HMG-CoA reductase increased the expression of CYP7A1 and altered clock gene expression such as BMAL1, PER2, and PER3 in the liver (74).

**Table 1:** The relationship between selective circadian related genes and pathogenic pathways in NAFLD.

Authors	Year	Study subjects	Genes related to circadian rhythm	Pathogenic pathways related to NAFLD	Main outcomes
**Shen et al.** (70)	2014	Mice	DEC1	SREBP-1c	DEC 1 negatively regulates hepatic SREBP-1c expression to reduce hepatic lipogenesis and TG content in liver.
**Iizuka et al.** (71, 72)	2008	Rats	DEC1	ChREBP	Glucose stimulation and overexpression of ChREBP increases the expressions of DEC1 and KLF-10, while overexpression of DEC1 or KLF-10 inhibits glucose stimulated lipogenesis *via* ChREBP.
	2011		KLF-10		
**Meng et al.** (73)	2020	Mice	XBP1	LCAT	Disruption of XBP1 impairs to PC-LPC cycle and fatty acids desaturation to promote the development of NAFLD *via* defecting the fatty acid monounsaturated and phospholipid remodeling pathways.
				LPCAT3	
				SCD1	
**Li et al.** (74)	2017	Mice	CYP7A1	HMG-CoA reductase	Inhibition of HMG-CoA reductase increased the expression of CYP7A1 and altered clock gene expression such as BMAL1, PER2, and PER3 in liver.
**Feng et al.** (21)	2011	Mice	REV-ERBα	HDAC3	REV-ERBα and HDAC3 co-localized near genes that regulate lipid metabolism. Loss of HDAC3 or REV-ERBα in the liver of mice leads to hepatic steatosis.
**Fleet et al.** (75)	2016	Mice	SRC-2	IGF1, ACLY, FASN et al.	Disruption of SRC-2 in mice led to a common comorbidity of metabolic syndrome also found in humans with NAFLD.

Dec1, Embryo-Chondrocyte-expressed Gene 1; KLF-10, Kruppel-like factor (KLF)-10; ChREBP, carbohydrate responsive element-binding protein; SREBP-1c, Sterol Regulatory Element-binding Protein-1c; XBP1, X-box binding protein 1; LCAT, lecithin–cholesterol acyltransferase; LPCAT3, lysophosphatidylcholine acyltransferase 3; SCD1, stearoyl-CoA desaturase 1; PC, phosphatidylcholine; LPC, lysophosphatidylcholines; CYP7A1, cholesterol 7-alpha-monooxygenase; HMG-CoA reductase, 3-hydroxy-3-methyl-glutaryl-coenzyme A reductase; HDAC3, histone deacetylase 3; SRC-2, steroid receptor coactivator-2; IGF1, Insulin-like growth factor 1; ACLY, ATP citrate lyase; FASN, Fatty Acid Synthase.

High-fat food intake and liver insulin resistance affect NAFLD *via* disruption of circadian clock (80, 81). If high-fat foods are ingested randomly, metabolic regulators will be disturbed, accompanied by weakened CREB oscillation, reduced AMPK activity, inhibited biological clock components (REV-ERBα, PER2), and elevated FASN expression. This resulted in increased long-chain free fatty acids in the liver (14, 45), at the levels of the synthesis, extension and/or desaturation of fatty acids.

Several genetic variations of clock genes are related to hepatic steatosis. Mice with circadian-related genes mutations underwent metabolic dysregulation and revealed more severe hepatic steatosis than did WT mice under conventional and high-fat food feeding conditions (82). Depletion of nuclear receptor REV-ERBα and REV-ERBβ in the liver disrupted clock genes and output genes such as CLOCK, BMAL1, CRY1, PER2, POR, PPARα, and SCO2. Moreover, liver-specific depletion of REV-ERBα and REV-ERBβ led to increased plasma glucose and TG levels and decreased FFAs levels (14). In addition, liver-specific depletion of both histone deacetylase 3 (HDAC3) and REV-ERBα in mice caused increases in hepatic TG levels (21). Finally, another study also showed that long-term deficiency of REV-ERBα activity led to moderate hepatic steatosis (76). Although the amount of HDAC3 is constant, its genome recruitment in the liver corresponds to the expression pattern of circadian rhythm REV-ERBα (21). The REV-ERBα binding site coincides with most HDAC3 binding sites at ZT10. Disruption of steroid receptor coactivator-2 (SRC-2), which regulates clock genes, resulted in hepatic steatosis. Furthermore, when circadian rhythm was chronically disrupted in SRC-2-/- mice, more severe hepatic steatosis phenotype was generated (75). In summary, the synthesis and decomposition of fat and TG are altered in patterns to promote fat accumulation in the liver when the circadian rhythm is dysregulated.

### Circadian Dysregulation Enhances Liver Inflammation

Although excessive accumulation of TG in hepatocytes is characteristic of NAFLD, steatosis alone is not necessarily pathogenic, because as in the early stages, NAFLD is reversible after weight loss and exercise. Oxidative stress, endoplasmic reticulum stress and the release of pro-inflammatory cytokines (such as tumor necrosis factor α, TNFα) are the main consequences of hepatic lipid overload, and are key factors in the progression of NAFLD to NASH. Free fatty acids and cholesterol, especially when accumulated in mitochondria, lead to increase TNFα and reactive oxygen species (ROS) production and play an early “inflammatory” role in promoting NASH. The increase in TNFα and ROS production due to excessive fat accumulation is known as lipotoxicity, which in turn causes inflammation, apoptosis, and consequently, the progression to hepatic fibrosis (62, 83). This process involves two alterations due to mitochondrial dysfunction including impared β-oxidation and endoplasmic reticulum stress, thereby resulting in lipid peroxidation. The resultant increase in ROS and the destruction of antioxidants’ activities are responsible for the initiation or exacerbation of inflammation (84). In the pathogenesis of NASH, hepatic resident cells (such as Kupffer cells and hepatic stellate cells) and cells recruited by injury (such as monocytes and macrophages) all release pro-inflammatory signals and participate in apoptosis or necrotic death of hepatocytes (85, 86). Infiltration of immune cells and the proinflammatory activation of immune cells are the two significant features of inflammation in NASH (87). The activation of nuclear factor kappa light chain enhancer of activated B cells (NF-κB) in hepatocytes, which is a nuclear transcription factor to regulate the expressions of inflammatory cytokines such as TNFα, interleukin 6 (IL-6), and interleukin 1β (IL-1β), leads to the recruitment and activation of Kupffer cells to mediate inflammation in NASH (88).

Much evidence suggests that circadian dysregulation is involved in the pathogenesis of inflammation in NASH (89). Recent studies have shown that inflammatory cytokines such as TNFα and IL-6 can resynchronize the circadian clock through activating NF-κB, which inhibits transcription of clock repressors (90, 91). In macrophages, BMAL1 increases the response of nuclear factor erythroid 2-related factor 2 (NRF2) to lipopolysaccharides (LPS) challenge, inhibiting the production of IL-1β (92). On the other hand. interleukin 10 (IL-10), an anti-inflammatory cytokine, is regulated by REV-ERBs throughout the circadian day (93). In a human study, HFD feeding altered cortisol rhythms, and brought about changes in diurnal oscillations of clock genes, inflammatory genes, and fat metabolic genes in monocytes (94). In addition, monocytes exhibit diurnal oscillations in the expression of clock genes (95).

Disruption of circadian rhythm (*via* using a chronic light-dark cycle shift paradigm) exacerbated the severity of HFD-induced inflammation, which was characterized by increases in the proinflammatory activation status and the expressions of inflammatory cytokines in bone marrow-derived macrophages (96). Myeloid-specific circadian disruption was reported to be sufficient to induce inflammatory responses. In myeloid-specific BMAL1 knockout mice, loss of BMAL1 led to disruption of rhythmic oscillations of clock genes such as ARNTL and nuclear receptor subfamily 1 group D member 1 (NR1D1), increased serum inflammatory cytokines such as IL-1β, IL-6, interferon gamma (INFγ), and monocyte chemoattractant protein-1 (MCP-1), and exacerbated metabolic dysregulation including increased adiposity and insulin resistance (95). Furthermore, myeloid cell-specific PER1/2 disruption *via* bone morrow transplantation worsened HFD-induced liver inflammation, which was accompanied with increased severity of hepatic steatosis and insulin resistance (97).

It suggested that the circadian disruption in macrophage is sufficient to bring about *in vivo* changes in hepatic steatosis and inflammation. SIRT1, an NAD^+^-dependent protein deacetylase, participates the maintenance of interaction between CLOCK and BMAL1 (52). Disruption of SIRT1 only in macrophage exacerbated HFD-induced hepatic steatosis *via* activating SREBP-1, activated hepatic fibrogenesis *via* stimulating collagen secretion, and elevated hepatic inflammation through activating NF-κB pathway and increasing hepatic macrophage infiltration (98).

**Figure 1:**
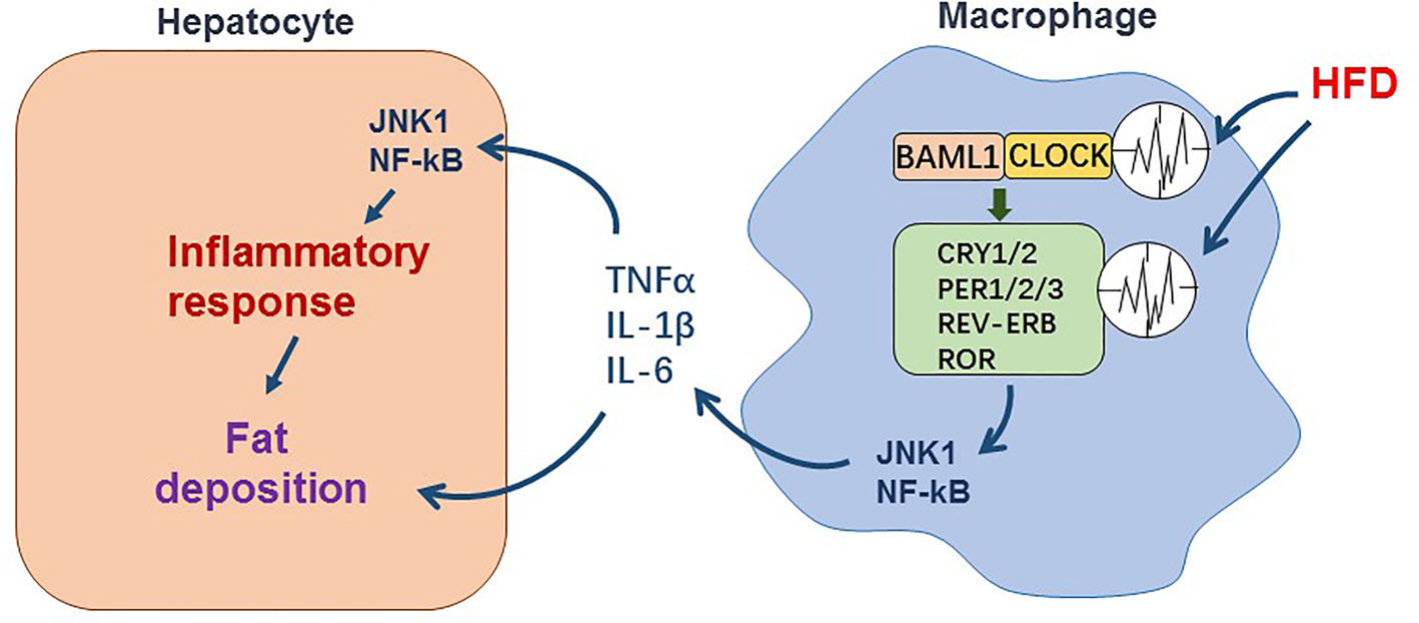
The effect of circadian dysregulation in macrophage induced by HFD on liver inflammation in NAFLD. Macrophage itself exhibits diurnal oscillations in expression of clock genes. During NAFLD, disruption of diurnal oscillations of clock genes induced by HFD promotes pro-inflammatory response in macrophage. The inflammatory cytokines such as TNFα, IL-1β, and IL-6 released by macrophage enhance inflammatory response and fat deposition in hepatocytes.

Collectively, circadian rhythm dysregulation resulted from high-fat diet appears to trigger chain reactions, which lead to activation of pro-inflammatory pathways in macrophages, excessive accumulation of fats in hepatocytes and liver inflammation (**Figure 1**). This underlies the pathogenesis of hepatic steatosis and inflammation in NAFLD.

### Distal Circadian Disruption Promotes NAFLD

Since adipose tissue and gut microbiota contribute significantly to NAFLD/NASH aspects including hepatic steatosis and inflammation, a “multiple hit hypothesis” has recently been proposed to better elucidate the pathogenesis of NAFLD and NASH. Adipose tissue is an endocrine organ which releases cytokines to influence other organs and regulate the systemic metabolism. A significant body of literature has validated that NAFLD is closely associated with the quantity, location and the types of fats in adipose tissue (99, 100). Moreover, visceral white fats appear be more critical in terms of promoting the development of NAFLD, although overall increased fat content is a risk factor for NAFLD (101, 102). During obesity, nutrition stress, e.g., nutrient overload or unhealthy nutrition, and environmental factors promote inflammation *via* activating macrophages and increasing macrophage infiltration into adipose tissue, leading to adipocyte dysfunction. The dysfunctional adipocytes reveal increased lipolysis and release a large amount of free fatty acids to distal tissues such as the liver. This in turn activates proinflammatory responses in hepatocytes and liver immune cells (103). Unhealthy diets and environmental factors also disturb gut microbiota, leading to increased gut permeability and release of gut microbiota metabolites and toxins such as LPS. These changes work collectively to increase fat synthesis and accumulation, as well as liver lipotoxicity. Mechanistically, lipotoxicity causes mitochondrial dysfunction and increases ROS production and ER stress, leading to hepatic inflammation and activation of liver fibrogenic program (104).

Adipokines, such as adiponectin, leptin, and resistin, are polypeptides produced by adipocytes, and are shown to contribute to NAFLD pathogenesis (105). In particular, adiponectin and leptin are capable of decrease liver fat accumulation and insulin resistance. Also, adiponectin is shown to reduce hepatic fibrosis and inflammation by blocking the activation of NF-κB and inhibiting the release of pro-inflammatory cytokines such as TNFα and IL-6 (106). In contrast, resistin have the opposite effects in the liver (107, 108). Leptin decreases fat accumulation in the liver and generally functions to improve insulin sensitivity, but promotes insulin resistance during leptin resistance states (108). During NAFLD, leptin increases liver inflammation and fibrosis through stimulating the production of transforming growth factor-β1 (TGF-β1) and activating hepatic stellate cells (109, 110). Adipokines, such as leptin and adiponectin oscillate within 24 hours to ensure the rational use of energy (111). Adiponectin was shown to regulate proinflammatory responses because its effect on increasing liver fat oxidation via inactivating ACC and activating AMPK. The latter likely involves increased expression of PPARα gene, which is controlled by circadian (28, 112). Adiponectin also is shown to reduce the activity of FASN, another enzyme involved in fatty acid synthesis (113). Environmental factor-mediated circadian rhythm disruption also amplified the pro-inflammatory responses of adipose tissue macrophages (114), leading to adipocyte dysfunction. The latter released a large amount of pro-inflammatory factors TNF α and IL-6 to inhibit adiponectin production (115–117). The diurnal oscillation of leptin secretion from adipose tissue mediated by C/EBPα was regulated by BMAL1/CLOCK. Disruption of circadian, for example, deletion of PER1/PER2 or under jet-lag, caused leptin resistance (118).

In addition, regulating the expression of genes in adipose tissue is shown to influence fat accumulation and inflammation in the liver. For instance, adipocyte-selective overexpression of PFKFB3/iPFK2, which activates 6-phosphofructo-1-kinase (6PFK1) to enhance glycolysis, decreased HFD-induced liver proinflammatory response and improved insulin signaling; although leading to increased hepatic steatosis (119–121). Also, disruption of interferon (α and β) receptor 1 (IFNAR1) only in adipose tissue exacerbated HFD-induced hepatic steatosis and systemic metabolic dysregulation (122).

The synthesis and storage of fats in adipose tissue reveal circadian rhythms (123). Deletion of adipose tissue BMAL1 disrupted circadian clocks in adipocytes and exacerbated HFD-induced obesity through increasing food intake during daytime and reducing energy expenditure. In addition, adipose tissue-specific BMAL1-disrupted mice exhibited decreases in a number of key enzymes involved in polyunsaturated fatty acid biosynthesis, affecting the energy metabolism homeostasis of the body (124). Moreover, CLOCK/BMAL1 was involved in fat formation and stimulation of lipid uptake by adipocytes *via* PPARγ activation (28, 125). PPARγ regulates the expression of REV-ERBα in adipocytes (126). Loss-of-PPARγ function in fat not only resulted in increases in FFAs and TG levels in plasma, but also elevated liver glucose production and insulin resistance (127, 128).

**Figure 2:**
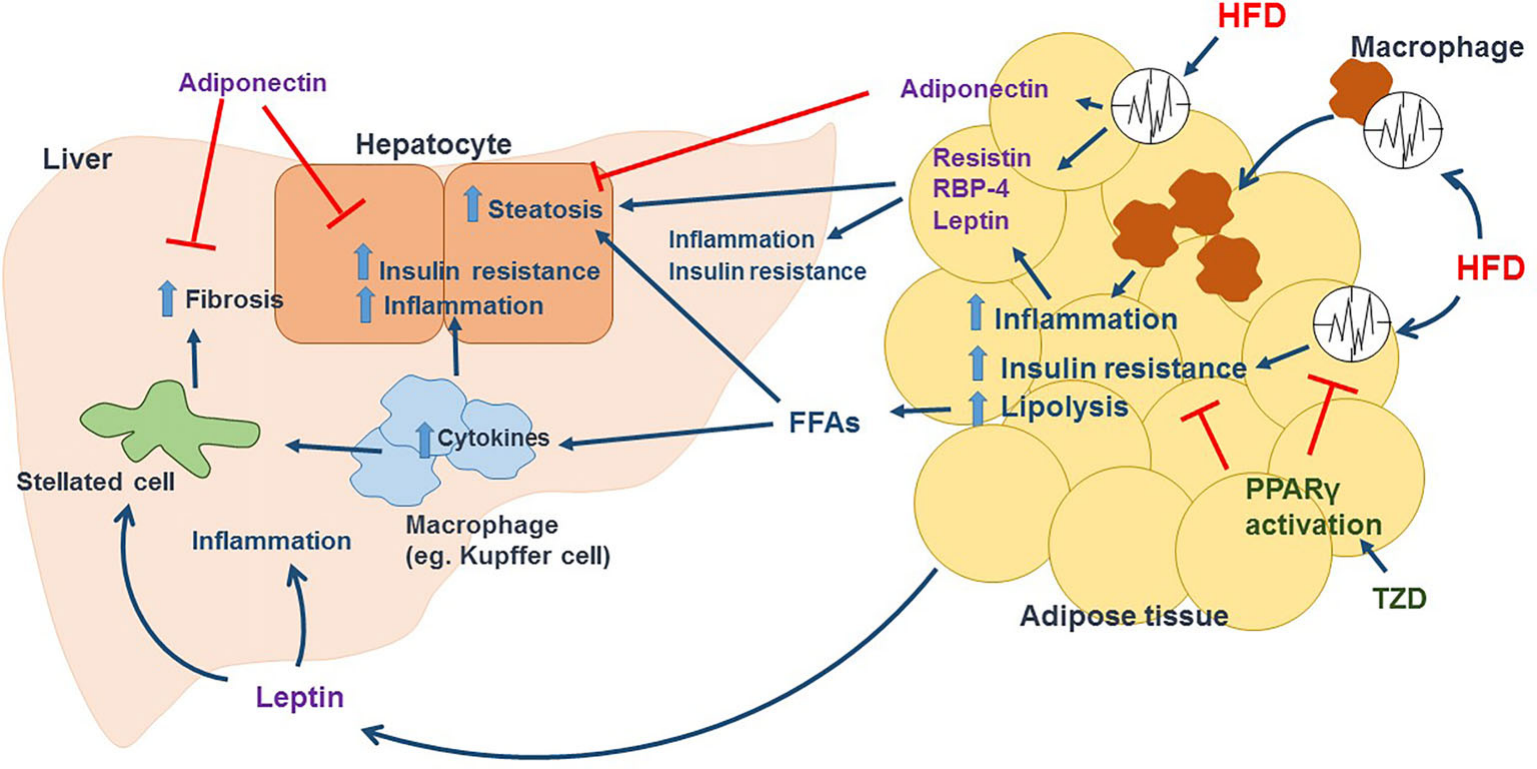
HFD induced circadian dysregulation in adipose tissue promotes the development of NAFLD. In adipose tissue, HFD disrupts circadian rhythm and promotes macrophage activation and infiltration, leading to inflammation and insulin resistance in adipocytes and cause adipocyte dysfunction. Lipolysis is enhanced in dysfunctional adipocytes, releasing large amount of FFAs in circulation, which increase fat accumulation in hepatocytes and activate immune cells such as Kupffer cells in liver. Activated immune cells release tons of inflammatory cytokines including TNFα and IL-6 to promote hepatic inflammation and insulin resistance. In addition, recruited immune cells and increased inflammation activate hepatic stellated cells, which cause collagen deposition and fibrosis. HFD also change the secretion of adipokines mediated by circadian clock. Dysfunctional adipocytes release Resistin and RBP-4, increasing steatosis, inflammation and insulin resistance in liver. HFD induces a leptin resistance condition, that leptin promotes inflammation, activates stellated cells and increase fibrosis in liver. Adiponectin, which can decrease hepatic steatosis, inflammation, insulin resistance, and fibrosis, is reduced in dysfunctional adipocytes induced by HFD. TZD such as rosiglitazone and pioglitazone activate PPARγ, restoring adipose tissue circadian rhythm and lowering inflammation and insulin resistance in adipose tissue, and further decreasing FFAs in circulation and the aspects of NAFLD/NASH.

Given what mentioned above and described in **Figure 2**, NAFLD is a metabolic disease of the liver. Circadian rhythm disorder accelerates the accumulation of excessive TG and promotes inflammation and abdominal obesity. Therefore, obese people with high visceral adipose tissue content reveal significantly increased incidences of NAFLD.

## Management of NAFLD/NASH Involving Circadian

To manage NAFLD, it is of a particular importance to control the prevalence of obesity, considering that obesity significantly promotes hepatic fat accumulation as a key characteristic of NAFLD. During the progression from NAFLD to NASH, inflammation plays a key role, and reducing inflammation can prevent liver damage and fibrosis. Lifestyle interventions and medications, which reduce weight and fat content, insulin resistance, and/or inflammation, are considered as effective ways to treat NAFLD. Although many medications were proved to be safe for NAFLD treatment, lifestyle interventions including dietary intervention and exercise are still the first choices. As such, patients are recommended to manage routine life according to the recommendations of healthy diet, loss of body weight, and exercise.

Caloric restriction, with or without exercise, was reported to reduce hepatic fat accumulation, inflammation and fibrosis, and to improve liver function through decreasing the serum levels of aspartate aminotransferase (AST) and alanine aminotransferase (ALT) in NAFLD (129, 130). However, BMAL1 knockout mice failed to increase life span under caloric restriction (131). In addition, peripheral circadian clock genes, timeless (TIM) and PER, were required for caloric restriction-mediated increase in lifespan and improvement in metabolic changes such as increased fat turnover and decreased TG synthesis in drosophila (132). Without caloric restriction, limiting feeding to certain times of the day that align with activity patterns is reported to reduce hepatic steatosis (45). Feeding an HFD within 8 hours per day (time-restricted feeding, TRF) is shown to protect against HFD-induced hepatic steatosis and liver damage. TRF also reduced HFD-induced elevation of hepatic gluconeogenesis by increasing CRY expression and suppressing CREB expression, leading to down-regulation of the expression of gluconeogenic genes such as pyruvate carboxylase and glucose-6-phosphatase (45). Compared to mice fed an HFD only during the 12 h dark phase, mice fed an HFD only during the 12 h light phase gain more body weight (44). This indicates the importance of timed feeding to maintaining weight or losing weight.

Mediterranean diet, which contains up to 40% of the calories from fat (mainly unsaturated fats) and 40% of the calories from carbohydrate (usually 50-60% in a low-fat diet), was reported to provide beneficial effects on NAFLD including reduction of liver fat and inflammation, as well as improvement of insulin resistance (133, 134). CLOCK gene polymorphisms were found to predict the loss of body weight of obese patients after consumption of Mediterranean diet (135). N-3 polyunsaturated fatty acids (PUFAs) were shown to reduce hepatic lipid synthesis and insulin resistance *via* regulating the expression of PPARα and SREBP-1, which were expressed with diurnally rhythms according to circadian clock (14, 136). Moreover, n-3 PUFAs also improved insulin signal pathway *via* modulating membrane fluidity and decreased proinflammatory responses *via* reducing the levels of TNF-α and IL-6 in NAFLD (133). Monounsaturated fatty acids (MUFAs) were known to suppress hepatic inflammation and ROS production, increase insulin sensitivity, and provide protective effects against NAFLD, although MUFAs might increase hepatic steatosis (137, 138). High carbohydrate diet and refined carbohydrates such as sucrose and fructose were found to promote the development of NAFLD through stimulating hepatic *de novo* lipogenesis and, thus, increase lipid accumulation in the liver (134). BMAL1 participated in hepatic *de novo* lipogenesis *via* the insulin-mTORC2-AKT signaling pathway as this was suggested by the findings that BMAL1 knockout mice exhibited decreased *de novo* lipogenesis in the liver and that *de novo* lipogenesis was recovered when restored AKT activity by insulin (139).

Sedentary lifestyle is a risk factor of the development of obesity and NAFLD. Exercise was found to improve insulin sensitivity in adipose tissue and decrease the flow of fatty acids to the liver, thereby reducing hepatic fat accumulation and insulin resistance. The effects of exercise on improving metabolic regulation were mediated by activation of AMPK, which inhibited hepatic lipogenesis through SREBP-1 to suppress the expression of lipogenic genes (such as ACC1 and FASN) and elevated hepatic fatty acid β-oxidation by reducing malonyl CoA and stimulating the expression of carnitine palmitoyltransferase 1 (CPT1) (140). A circadian phosphoproteome study in mouse liver indicated that 25% of phosphopeptides including AKT, AMPK and mTOR oscillated and signaling pathways for metabolism were regulated rhythmically by phosphorylation (141). Choosing the right time of the day to exercise was shown to obtain optimal beneficial effects due to that hypoxia-inducible factor 1a (HIF1a), a transcription factor responsible for regulating glycolysis under low oxygen condition, was activated in a time-dependent manner upon exercise (142). In addition to exercise, sleep-awake cycle plays an important role in maintaining metabolic homeostasis as indicated by the finding that late bedtime was positively associated with the incidence of NAFLD in human (143).

There are several pharmaco-therapeutic options for NAFLD/NASH. Thiazolidinediones (TZDs), which induce PPARγ activation, are considered an effective medication to increase insulin sensitivity, reduce adipose tissue inflammation and improve biopsy parameters of steatohepatitis (144). For example, pioglitazone was reported to reduce liver fibrosis and adipose tissue insulin resistance in NASH patients with and without type 2 diabetes, but more effective in patients with type 2 diabetes (145). Rosiglitazone, a ligand of PPARγ, recovered HFD-induced the changes of hepatic BMAL1 function, which increased the recruitment and activity of BMAL1 target genes, such as DBP, CHRONO, and FABP2, and increased liver insulin sensitivity as well (146). Glucagon-like peptide 1 (GLP-1), which is a peptide hormone secreted by intestine L cell after food intake, stimulates insulin release and inhibits glucagon release, thereby improving glucose homeostasis. The secretion of GLP-1 was diurnally oscillated and paralleled with the expression of BMAL1, which was suppressed by palmitate (147). GLP-1 receptor (GLP-1R) agonism was found to reduce body weight and hepatic steatosis, as well as increased hepatic fatty acid oxidation and insulin sensitivity in NAFLD/NASH (148). Dipeptidyl peptidase-4 (DPP-4) inhibitors suppressed DPP-4 enzyme, thereby preventing inactivation of GLP-1 and glucose-dependent insulinotropic polypeptide (GIP) to stimulate insulin secretion. DPP-4 inhibitors also were reported to decrease body mass index, liver triglyceride levels, serum aminotransferase levels, and the progression of NAFLD (149). Blocking sodium-glucose cotransporter 2 (SGLT2) inhibited glucose absorption. Also, SGLT2 inhibitors reduced body weight, blood pressure, and insulin resistance, which is considered a potential medication for NAFLD/NASH. Metformin, a first-line anti-diabetic drug, was shown to provide beneficial effects in patients with NAFLD/NASH including reduction of serum lipids and glucose levels. However, the improvement of liver histology and function by metformin was not significant (150). The anti-diabetic effect of metformin also was considered to be mediated by gut microbiota, which was supported by the study that transferring fecal samples from metformin-treated mice to germ-free mice improved glucose tolerance (151). Gut microbiota has its own diurnal compositional and functional oscillations. Nutrition and environmental factors such as HFD and disruption of feeding time are shown to impair microbiota diurnal rhythmicity and cause microbiota dysbiosis, leading to metabolic dysregulation (152). Obeticholic acid (OCA), which is a synthetic bile acid derivative and farnesoid X receptor agonist, was recently found to reduce hepatic inflammation and improve the histological features of NASH (153). This was attributable to alteration of gut microbiota composition by increased population of *Blautia* and decreased levels of taurine-bound bile acid induced by an HFD after OCA treatment (154). Also, patients with NAFLD are in a need to consider the time and intervals of taking certain drugs, given that liver detoxification is affected by biological rhythms.

## Conclusion and Future Directions

Nutrition and lifestyle are known to affect metabolism, thereby profoundly influencing human health, quality of life and longevity. Circadian clock plays an important role in maintaining metabolic homeostasis. Dysregulation of circadian rhythms induced by high-fat and/or high-sugar diets and unhealthy eating patterns such as eating throughout the day and late-night eating are considered pathogenic factors for NAFLD/NASH. As summarized in this review, we provide the current state of knowledge for NAFLD pathogenesis from a circadian perspective. However, the pathogenesis of NAFLD/NASH is multifactorial and complex, which makes it difficult to develop an effective therapy for treating NAFLD/NASH. Dietary and lifestyle interventions, which promote weight loss, are still considered effective to improve NAFLD/NASH aspects.

## Author Contributions

XG and JZ wrote the manuscript. SZ, XJ, TC, and JY participated in revision. SW, XM, and CW organized the manuscript. XG and CW came up with the concept. All authors contributed to the article and approved the submitted version.

## Funding

The development of this review was supported by The Fundamental Research Funds of Shandong University (Grant No. 2017TB0028), Young Scholars Program of Shandong University (Grant No. 2018WLJH33), National Natural Science Foundation of China (Grant No. 81803224) and National Natural Science Foundation of China (Grant No. 81770772).

## Conflict of Interest

The authors declare that the research was conducted in the absence of any commercial or financial relationships that could be construed as a potential conflict of interest.

## Publisher’s Note

All claims expressed in this article are solely those of the authors and do not necessarily represent those of their affiliated organizations, or those of the publisher, the editors and the reviewers. Any product that may be evaluated in this article, or claim that may be made by its manufacturer, is not guaranteed or endorsed by the publisher.
